# Immune Checkpoints as a Target for Colorectal Cancer Treatment

**DOI:** 10.3390/ijms18061324

**Published:** 2017-06-21

**Authors:** Alessandro Passardi, Matteo Canale, Martina Valgiusti, Paola Ulivi

**Affiliations:** 1 Department of Medical Oncology, Istituto Scientifico Romagnolo per lo Studio e la Cura dei Tumori (IRST) IRCCS, 47014 Meldola, Italy; alessandro.passardi@irst.emr.it (A.P.); martina.valgiusti@irst.emr.it (M.V.); 2 Biosciences Laboratory, Istituto Scientifico Romagnolo per lo Studio e la Cura dei Tumori (IRST) IRCCS, 47014 Meldola, Italy; matteo.canale@irst.emr.it

**Keywords:** colorectal cancer, immune checkpoint inhibitors, microsatellite instability (MSI), programmed death-1 receptor (PD1), programmed cell death protein ligand 1 (PD-L1)

## Abstract

Anti-tumor immunity is a new line of research for the treatment of patients with solid tumors. In this field, negative regulators of the immune system called immune checkpoints play a key role in limiting antitumor immunologic responses. For this reason, immune checkpoint-inhibiting agents, such as those directed against cytotoxic T-lymphocyte antigen 4 (CTLA-4) and programmed death-1 receptor (PD1) and its ligand PD-L1, have been developed as antitumor drugs, producing interesting results in preclinical and clinical studies. We present an updated review of the biological background and clinical development of immune checkpoint inhibitors in colorectal cancer (CRC). Early trial results on PD1 and PD-L1 blockade appear promising, especially in CRC patients with microsatellite instability (MSI). Clinical trials are ongoing to confirm these preliminary results, evaluate combination strategies and identify biomarkers to predict which patients are most likely to benefit from, or show resistance to, the effects of checkpoint inhibition.

## 1. Introduction

Colorectal cancer (CRC) is the second leading cause of cancer-related death. Recent therapeutic approaches that add epidermal growth factor receptor (EGFR) and vascular endothelial growth factor (VEGF) targeted agents to standard chemotherapy have produced a prolonged overall survival (OS) of up to 30 months in patients with metastatic disease [1]. However, research is ongoing to further improve the outcome of metastatic CRC (mCRC) patients.

The relationship between cancer cells and host immune cells in the tumor microenvironment has been the object of growing interest among researchers, and cancer cell escape from the immune system response was recently recognized as an independent hallmark of cancer [2]. Immunotherapy has made numerous advances in this area because of a better understanding of immuno-oncology. Immune checkpoint inhibitors are one of the most promising immunotherapy approaches, obtaining U.S. Food and Drug Administration (FDA) approval for the treatment of different advanced malignancies such as melanoma and non-small cell lung cancer [3]. On the basis of these encouraging results, trials are also ongoing for patients with mCRC.

This paper presents a description of the interconnection between tumor and immune system, together with a summary of the immunological features of CRC and an up-to-date overview of the role of checkpoint inhibitors in mCRC.

## 2. The Immune System and the Tumor

The response of the immune system against the tumor is a concept known as cancer immunosurveillance [4,5]. It is generally accepted that the immune system, through its innate and adaptive effector mechanisms, is capable of recognising and eradicating transformant cells in the early stages of carcinogenesis.

Conversely, there is increasing evidence that the immune system plays an active part in the development of the tumor, not only as a facilitator of cell transformation but also as a promoter of uncontrolled growth and modulator of immunogenicity [6]. The potential role of the immune system in tumor transformation was initially suggested by Virchow who, observing the lymphoreticular infiltrates surrounding malignant tumors, hypothesized that chronic inflammation of healthy tissues may induce and facilitate cancerogenesis [7].

Modulation of the tumor response by cancer cells through mechanisms still to be elucidated is referred to as immunoediting [8]. This process, a delicate balance between cancer cells and the immune system within the tumor microenvironment, is divided into three phases: the elimination phase, equilibrium phase and escape phase.

**Figure 1:**
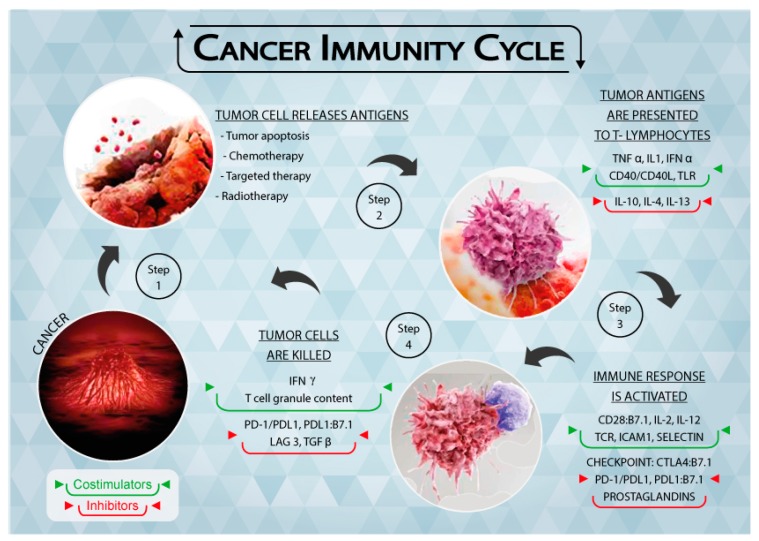
Schematic representation of the Tumor Immunity Cycle. As a consequence of apoptosis and anti-tumor treatments, cancer cells release tumor antigens (Step 1) that are presented to T lymphocytes through the major histocompatibility complex (MHC) (Step 2). The immune response is thus activated, together with immune checkpoints that regulate the extent of the response (Step 3). Cancer cells are recognized and killed by activated T lymphocytes (Step 4), causing them to release further tumor antigens. Figure 1 is complete ownership of authors.

In the elimination phase, the immune system is able to detect and eradicate tumor cells, which is generally referred to as cancer immunosurveillance [9]. This phase follows the “two-signal” theory, i.e., tumor antigens are presented to the T cell receptor (TCR) through the major histocompatibility complex (MHC) (first signal) and the subsequent binding of co-stimulators leads to the activation of T lymphocytes (second signal). Both adaptive and innate immunity responses contribute to the definition of the process (Figure 1), resulting in efficient tumor prevention by the immune system.

In the equilibrium phase, cancer cells activate several biochemical pathways to negatively regulate the immune response, reaching a dynamic state of immune tolerance. However, the exhausted effector T lymphocytes are no longer efficient at tumor suppression. The equilibrium phase is considered to be the longest stage of the cancer immunoediting process in which immune cells, still active within the tumor microenvironment, keep the cancer cells in a state of dormancy but are not capable of eradicating them [10]. This dormant and clinically non-relevant phase of malignancy lasts until the escape phase begins and the cancer cells, taking advantage of their heterogeneity and genetic variability, establish several mechanisms to protect themselves from immune effector functions [4]. In this phase, the tumor is tolerated by the immune system and the cancer cells also suppress immune responses directly and indirectly exploiting the physiological pathways that serve to maintain tissue homeostasis and prevent normal tissue damage and autoimmunity through multiple mechanisms [11]. In particular, the cancer cells bind and activate the co-inhibitory molecules on the T lymphocyte surface, e.g., T lymphocyte-associated antigen 4 (CTLA4), programmed cell death protein 1 (PD1), lymphocyte activation gene 3 (*LAG3*) and T cell immunoglobulin mucin 3 (TIM3). They also express inhibitory co-receptors such as programmed cell death protein ligand 1 (PD-L1), secrete soluble immunosuppressive mediators such as indoleamine 2,3-dioxygenase (IDO), and contribute to the release of anti-inflammatory interleukins such as transforming growth factor-β (TGF-β) and interleukin 10 (IL-10) into the tumor microenvironment [12,13,14]. These co-inhibitory molecules, known as immune checkpoints, play a crucial role in blocking the host immune response.

Given the ability of the tumor to modulate the immune response through these immune response mediators, research has recently focused on understanding whether immune checkpoint-targeted drugs can interrupt the inhibition of the immune signal against tumors and restore the antitumor efficiency of the immune system [15]. We will now proceed to analyze the role of these molecules in the anti-tumor response.

## 3. Immune Checkpoints

Recent immunotherapeutic strategies based on the modulation of the immune response aim to increase the activation of the functional effectors of T cells, leading to an amplification of the immune response [3]. The main targets of this strategy are CTLA4 and PD1 and its ligand, PD-L1. CTLA4 (CD152) is a membrane glycoprotein that closely resembles CD28, binding the same ligands of the B7 family (CD80 and CD86) on the surface of antigen-presenting cells (APC). Following antigenic stimulation of the TCR, the T cell acquires the capacity to express CTLA4 which binds B7 molecules with higher affinity than CD28. Unlike CD28/B7, which activates cytotoxic immunity, the CTLA4/B7 interaction inhibits the T response and plays an important role in the maintenance of immune tolerance [11]. Moreover, CTLA4 expressed by immunosuppressive T regulatory cells (Treg) generates the downregulation of B7 molecules on the APC surface, contributing to negatively modulating the T cell effector action [16]. In preclinical studies, the blockade of CTLA4 led to a 1.5- to 2-fold increase in the proliferation of T cells, a 6-fold increase in the production of interleukin-2 [17,18] and the depletion of T regulatory lymphocytes in the tumor microenvironment through a macrophage-dependent process [18,19].

PD1 (CD279) is an inhibitory co-receptor expressed on the cell surface of T lymphocytes CD8^+^ and CD4^+^, natural killer cells (NK), B lymphocytes and tumor-infiltrating lymphocytes (TILs) [14]. It plays a key role in balancing tumor immunity and inflammatory reactions, thus attenuating the late immune response mediated by T lymphocytes that have migrated to the tumor microenvironment. In normal tissues this mechanism prevents repeated and protracted tissue insult that causes irreversible damage [11,15,20].

PD1 interacts with 2 ligands: PD-L1 (CD 274), expressed on the cell surface of activated lymphocytes (T, B and NK) [3], peripheral tissues and organs [16], and to a greater extent by tumor cells, and PD-L2, expressed primarily by macrophages and dendritic cells [21]. The expression of PD1 by exhausted T cells indicates their lost capability to execute their effector function, while the bond between PD1 and PD-L1/2 leads to the inhibition of T cell activation and cytokine secretion, i.e., interferon-γ (IFN-γ), tumor necrosis factor-α (TNF-α) and interleukin 2 (IL-2), and helps to maintain immune homeostasis by avoiding the onset of autoimmunity [22]. In this setting, PD-L1 expressed by cancer cells is part of a mechanism called adaptive immune resistance in which tumor and stromal cells downmodulate the infiltrating T cells in the tumor microenvironment [3]. In preliminary evaluations, tumor PD-L1 expression was associated with a poor prognosis in several tumor types and was considered to play a major role in processing the immune response against the tumor. Some studies have refuted this hypothesis, documenting a favorable outcome in patients with PD-L1-positive melanoma [23]. Moreover, blocking the bond between PD1 and PD-L1 may lead to the reactivation of cytotoxic T lymphocytes, restoring their ability to attack cancer cells.

## 4. Immunological Features of CRC

CRC is divided into sporadic, familial or hereditary on the basis of its etiology. Sporadic CRC accounts for about 70–75% of all cases of CRC and is mainly linked to environmental lifestyle risk factors such as diet, obesity, smoking and alcohol consumption, whereas the germline component does not seem to be involved [24]. In contrast, expected or known germline alterations are involved in familial and hereditary CRC. Familial CRC accounts for about 20% of all CRCs and is characterized by a positive family history, but a causative germline alteration has yet to be identified [25]. Conversely, high-penetrant predisposing germline mutations are observed in hereditary CRC, which accounts for about 5–10% of CRC [25,26]. Lynch syndrome is an autosomal dominant inherited syndrome caused by monoallelic germline alterations of the DNA mismatch repair (MMR) genes such as MSH2, MLH1, MSH6 and PMS2 [27]. Defects in the DNA MMR pathway play a crucial role in the development of CRC patients with Lynch syndrome. In fact, in MMR-proficient cells, MMR proteins recognize and correct different types of misincorporations, insertions and deletions introduced by DNA polymerase slippage. These replication errors are especially frequent in repetitive DNA sequences such as microsatellites [28]. Consequently, microsatellite instability (MSI) develops in MMR-deficient cells and virtually all CRC derived from Lynch syndrome patients have MSI [29].

MSI is also observed in about 15–20% of sporadic CRC [30] and is associated with a better prognosis [31]. In this setting, the most frequent molecular mechanism responsible for MSI is the biallelic inactivation of MLH1 by promoter hypermethylation [32], commonly associated with a methylator phenotype. Overall, the somatic mutations that drive tumorigenesis following the inactivation of the DNA MMR pathway seem to be comparable in sporadic and familial MSI CRC. Furthermore, it has been shown that about 1300 somatic mutations are acquired in MSI CRC derived from patients with Lynch syndrome, whereas an average of only 190 mutations are present in tumors with microsatellite stability (MSS) [33]. A high number of mutations is associated with increased tumor immunogenicity due to an elevated production of neoantigens [34]. Moreover, a high number of tumor-infiltrating lymphocytes (TILs) have been observed in CRC and especially in MSI tumors [35], which could, in part, be explained by an upregulation of CD103 in CD8+ cells in MSI compared to MSS tumors [36]. A high concentration of granzyme B and perforin has been found in CD8+ T cells in MSI tumors, which is responsible for their reactive status [37,38]. Dense CD4+ T cell infiltration has also been observed in MSI tumors [39]. Furthermore, tumor dendritic cells in MSI patients have been shown to express higher levels of co-stimulatory molecules than those of MSS tumors [37].

Within this context, patients with MSI-high (MSI-H) tumors evaluated by MMR protein loss, immunohistochemistry or PCR, represent a subgroup more likely to benefit from immune checkpoint inhibitors. Repair system deficiency leads to an increase in somatic mutations which, in turn, increases immunogenicity. Similarly, a high tumor burden or mutations in the proofreading domain of the DNA polymerase POLE appears to be more responsive to anti-PD1 therapy [40].

Conflicting results are present in the literature with regard to PD-L1 expression in CRC in relation to MSI. Some studies have reported a higher expression of PD1 and PD-L1 in high MSI (MSI-H) CRC than in MSS tumors [41,42]. These data were recently confirmed by Inaguma et al. [43] who reported high PD-L1 expression in BRAF-mutated MMR-deficient tumors, typically located in the right or transverse colon. Conversely, Droeser et al. observed a higher frequency of PD-L1 expression in patients with MSS tumors [44]. A recent study by Li et al. did not reveal any significant differences in PD1 and PD-L1 expression between MSI-H and MSS CRC patients, but reported that a higher expression of TIL PD1 and tumor PD-L1 was associated with better prognosis, especially in patients with MMR-proficient disease [45].

These observations, together with the knowledge that CRC is one of the tumor types with the highest mutation prevalence and, consequently, with the highest antigenic potential [46], have led to the development of trials studying the therapeutic efficacy of immune checkpoint inhibitors. Ongoing phase 2 and 3 trials with immune checkpoint inhibitors are listed in Table 1.

## 5. Clinical Results of Immune Checkpoint Inhibitors in mCRC

Since the treatment efficacy of checkpoint inhibitors was demonstrated in malignant melanoma, renal cell cancer and lung cancer, several trials have been conducted on solid tumors, in particular, mCRC. We report here the updated results of published phase I and II trials of anti CTLA4, anti PD1 and anti PD-L1 agents in mCRC.

### 5.1. Cytotoxic T-Lymphocyte-Associated Antigen (CTLA)-4 Blockade

Ipilimumab and tremelimumab are CTLA-4 inhibitors currently under development for use in humans. Tremelimumab is a fully human anti CTLA4 IgG 2 monoclonal antibody (MAb). A single-arm multicenter phase II trial of intravenous tremelimumab every 90 days was conducted on 47 mCRC patients after failure of all standard chemotherapeutic treatments [47]. Patients were not selected on the basis of MMR status or MSI. Study results were not suggestive of a significant activity of tremelimumab as a single agent in this population. Treatment was well tolerated with grade ≥3 toxicities in 19.1% of patients. Only one patient received the second treatment dose and achieved a partial response lasting for six months. Median OS was 19.1 months and median progression-free survival (PFS) was 2.3 months. These results do not encourage further research into the CTLA4 blockade in mCRC, at least in an unselected population.

### 5.2. Programmed Death (PD1) Blockade

Nivolumab is a fully humanized IgG4 MAb directed against PD1. The drug was initially investigated in a phase I trial of patients with solid tumors, with four dose escalation cohorts (0.3, 1, 3 and 10 mg/kg) [48]. Among 39 enrolled patients, 14 had mCRC. A durable complete response off-treatment was reported in one patient with CRC. B7-H1 expression on the tumor cell surface was indicated as possible predictor marker of response. Although the drug was further evaluated in a phase II trial on 296 patients with various solid tumors, no objective responses were found among those with mCRC [49]. As the only responder among mCRC patients was found to have a deficient MMR (dMMR) tumor, this status was presumed to be a predictor of efficacy. CheckMate-0142 is a phase 2 study investigating the efficacy and safety of nivolumab ± ipilimumab in patients with either MSI-H or non-MSI-H mCRC [50]. The primary endpoint is investigator-reported objective response rate (ORR). At the 2017 Gastrointestinal Cancers Symposium, updated results on 74 patients treated in the monotherapy arm were presented (the combination arm is still enrolling). The majority of patients (54%) had received at least three prior lines of chemotherapy. BRAF and KRAS were mutated in 16% and 35% of patients, respectively; 28% of patients had PD-L1 positive tumors and 31% had Lynch syndrome. The objective response rate per investigator assessment was 31.1% and the disease control rate was 68.9%. Objective responses were observed regardless of PD-L1 expression, BRAF and KRAS mutation status, or the presence of Lynch syndrome. Median PFS was 9.6 months and median OS had still not been reached.

Pembrolizumab is a humanized IgG4 MAb directed against PD1 receptors. The drug was investigated in a phase I trial on 32 patients with advanced solid tumors, including three patients with mCRC, all of whom showed early progression and discontinued treatment [51]. The hypothesis of dMMR status as a predictor of efficacy of PD1 inhibitors was tested in a phase 2 study of pembrolizumab on patients with dMMR mCRC, mismatch repair-proficient (pMMR) mCRC, and dMMR cancers other than those of the colorectum [52,53,54,55]. Pembrolizumab was administered at 10 mg/kg every 14 days in patients previously treated for metastatic disease. The immune-related objective response rate and immune-related PFS rate at 20 weeks (primary endpoints of the study) were 40% and 78%, respectively, for dMMR CRC and 0% and 11% for pMMR mCRC. Moreover, the median PFS and OS were not reached in the cohort with dMMR CRC but were 2.2 and 5.0 months, respectively, in the pMMR mCRC cohort (hazard ratio for disease progression and death, 0.10 [*p* < 0.001], and 0.22 [*p* = 0.05], respectively). Whole-exome sequencing revealed the presence of a mean of 1782 somatic mutations in the dMMR cohort vs. only 73 in MMR-proficient tumors (*p* = 0.007). Moreover, high somatic mutation loads were associated with prolonged OS (*p* = 0.02). These results seem to confirm that a dMMR status is predictive of pembrolizumab efficacy.

The efficacy of pembrolizumab was recently evaluated in 149 patients with MSI-H/dMMR cancer enrolled in five open-label, single-arm trials [52,53,54,55,56]. Results from these trials prompted the FDA to grant accelerated approval to pembrolizumab for the treatment of (1) adult and pediatric patients with unresectable or metastatic MSI-H or dMMR refractory solid tumors for whom there are no alternative treatment options; and (2) patients with MSI-H or dMMR CRC who become resistant to fluoropyrimidine, oxaliplatin and irinotecan. In particular, among 90 patients with CRC, the objective response rate (ORR) was 36% (95 CI 26–46%) lasting from 1.6 to 22.7 months.

### 5.3. Programmed Death-Ligand 1 (PD-L1) Blockade

BMS936559 (MDX 1105), a fully human anti PD-L1 MAb, was tested in a phase I/II study on more than 200 patients with various solid tumors (including 18 CRC patients). Although a 17% objective response was reported, none were seen in CRC [57].

Atezolizumab is a humanized PD-L1-targeting IgG1 MAb that inhibits binding to both PD1 and B7.1 to enhance T-cell priming and reinvigorate suppressed immune cells [58,59]. Although monotherapy activity has been demonstrated in solid tumors, response rates in MSS CRC are not encouraging. An open-label, multicenter phase Ib study was conducted to investigate the activity of atezolizumab in combination with bevacizumab in 14 patients with refractory mCRC (Arm A) and with bevacizumab + FOLFOX in 30 oxaliplatin-naive mCRC patients (Arm B) [60]. Arm A patients received atezolizumab 20 mg/kg q3w and bevacizumab 15 mg/kg q3w, while Arm B patients received atezolizumab 14 mg/kg q2w, bevacizumab 10 mg/kg q2w and FOLFOX at standard doses. The ORR was 8% (1/13) in Arm A and 36% (9/25) in Arm B. The ORR was 44% (8/18) for Arm B first-line patients.

In preclinical models, targeted inhibition of MEK leads to upregulation of major histocompatibility complex (MHC) I in tumor cells, induces intratumoral T-cell infiltration and enhances anti-PD-L1 activity [61]. A phase Ib study evaluating the combination of the MEK inhibitor cobimetinib and atezolizumab was carried out in patients with advanced solid tumors. Cobimetinib was escalated from 20 to 60 mg daily (21 days on/7 days off) and combined with atezolizumab 800 mg administered intravenously every two weeks. Twenty-three mCRC patients were enrolled, no dose-limiting toxicities were observed, and an expansion cohort was treated with atezolizumab 800 mg and cobimetinib 60 mg. The ORR was 17% and was not influenced by baseline PD-L1 expression. Among the four responders, three had pMMR tumors and one had an unknown MMR status. Results from serial biopsies showed enhanced PD-L1 upregulation, CD8 T-cell infiltration and MHC I expression after treatment, providing a strong rationale for the combination.

## 6. Conclusions

Despite continuous improvements in multidisciplinary treatment, mCRC remains a major cause of death. Within this context, immunotherapy could play an important role to further improve patient outcome. A better understanding of the interaction between the tumor and the immune system over the past few decades has led to the development of new agents, in particular, checkpoint inhibitors.

Treatment efficacy of checkpoint inhibitors was initially demonstrated in tumors with high mutational burden, malignant melanoma in primis, but also renal cell cancer and non-small cell lung cancer. Following the successful development of the drugs for these malignancies, several trials have been conducted in other solid tumors, including CRC. Approximately 4% of mCRC patients present with MSI-H, which indicates a dMMR system. This condition is associated with an increased mutational burden and immune cell infiltration, making these patients ideal candidates for immune checkpoint inhibitors. Clinical trials are ongoing in both dMMR and pMMR mCRC.

One important limitation of this review is that we only had exploratory data from phase I and II trials at our disposal given that randomized controlled trials in large patient cohorts are still lacking. Trials with anti-CTLA4-directed agents did not show significant activity and further development of this class of drug in mCRC is not encouraged, at least in patients with pMMR status. Conversely, early trial results on PD1 blockade (nivolumab and pembrolizumab) appear promising, especially in patients with dMMR mCRC. The FDA’s accelerated approval of pembrolizumab to treat MSI-H or dMMR refractory solid tumors is expected to further attract the attention of the scientific community to this topic.

Moreover, despite the weak activity of the PD-L1 blockade as monotherapy, trials of atezolizumab in combination with both bevacizumab and cobimetinib have opened the way to combination strategies, which could extend the indication of immune checkpoint inhibitors to pMMR mCRC. Similarly, early trial results on the combination of nivolumab and ipilimumab have shown encouraging clinical activity and survival in MSI-H mCRC patients.

The identification of predictive markers is of the utmost importance in this clinical setting. A better understanding of genomic features related to dMMR status could help us to find more selective markers to predict the efficacy of immune checkpoint inhibition, e.g., checkpoint proteins or TILs. Given that the vast majority of mCRCs show microsatellite stability, predictive markers are also needed in this subgroup to select patients who are most likely to benefit from immunotherapy.

## Figures and Tables

**Table 1:** Ongoing phase II and III clinical trials on immune checkpoint inhibitors in mCRC.

ClinicalTrials.gov Identifier	Agent	Trial	Patient Population	Phase	Primary Endpoint
NCT02860546	Nivolumab	A study evaluating TAS-102 plus nivolumab in patients with MSS CRC	mCRC	2	irORR
NCT02060188	Nivolumab	An investigational immunotherapy study of nivolumab and nivolumab in combination with other anti-cancer drugs in colon cancer that returned or spread (CheckMate142)	MSI/MSS mCRC	2	ORR
NCT02981524	Pembrolizumab	Phase 2 Study of GVAX (With CY) and pembrolizumab in pMMR advanced colorectal cancer	MMR-p mCRC	2	ORR
NCT02437071	Pembrolizumab	Assessment of the efficacy of pembrolizumab plus radiotherapy or ablation in metastatic colorectal cancer patients	mCRC	2	ORR
NCT02563002	Pembrolizumab	Study of pembrolizumab (MK-3475) vs. standard therapy in patients with microsatellite instability-high (MSI-H) or mismatch repair deficient (dMMR) stage IV colorectal carcinoma (MK-3475-177/KEYNOTE-177)	mCRC	3	PFS
NCT01876511	Pembrolizumab	Phase 2 Study of MK-3475 in patients with microsatellite instability (MSI) tumors	MSI/MSS mCRC	2	irORR/irPFS
NCT02788279	Atezolizumab	A study to investigate efficacy and safety of cobimetinib plus atezolizumab and atezolizumab monotherapy vs. regorafenib in patients with metastatic colorectal adenocarcinoma	mCRC	3	OS
NCT02291289	Atezolizumab	A multi-center study of biomarker-driven therapy in metastatic colorectal cancer	mCRC	2	PFS
NCT02992912	Atezolizumab	Atezolizumab with stereotactic ablative radiotherapy in patients with metastatic tumors (SABR-PD-L1)	Metastatic tumors	2	PFS
NCT03050814	Avelumab	Standard of care alone or in combination with Ad-CEA vaccine and avelumab in patients with previously untreated metastatic colorectal cancer (QUILT-2.004)	mCRC	2	18mPD
NCT02870920	Tremelimumab	Durvalumab and tremelimumab and best supportive care vs. best supportive care alone in patients with advanced colorectal adenocarcinoma refractory to standard therapies	mCRC	2	OS
NCT02227667	MEDI4736	Evaluation of the efficacy of MEDI4736 in immunological subsets of advanced colorectal cancer	mCRC	2	BRR

mCRC, metastatic colorectal cancer; MSI, microsatellite instability; MSS, microsatellite stability; MMR-p, mismatch repair proficient; ORR, objective response rate; irORR, immune-related ORR; PFS, progression-free survival; irPFS, immune-related PFS; OS, overall survival; 18mPD, progressive disease at 18 months; BRR, best response rate. Details available at: www.clinicaltrials.gov.

## Abbreviations

CTLA-4	Cytotoxic T-lymphocyte antigen 4
PD1	Programmed death-1 receptor
PD-L1	Programmed death-1 receptor ligand
MMR	Mismatch repair
CRC	Colorectal cancer
